# A High-Performance LC Wireless Passive Pressure Sensor Fabricated Using Low-Temperature Co-Fired Ceramic (LTCC) Technology

**DOI:** 10.3390/s141223337

**Published:** 2014-12-05

**Authors:** Chen Li, Qiulin Tan, Chenyang Xue, Wendong Zhang, Yunzhi Li, Jijun Xiong

**Affiliations:** 1 Key Laboratory of Instrumentation Science & Dynamic Measurement, Ministry of Education, North University of China, Tai Yuan 030051, China; E-Mails: flanklichen@163.com (C.L.); wdzhang@nuc.edu.cn (W.Z.); lyz111@nuc.edu.cn (Y.L.); 2 Science and Technology on Electronic Test & Measurement Laboratory, North University of China, Tai Yuan 030051, China; E-Mail: xuechenyang@nuc.edu.cn

**Keywords:** wireless passive pressure sensor, LTCC technology, high temperature characterization, high pressure characterization

## Abstract

An LC resonant pressure sensor with improved performance is presented in this paper. The sensor is designed with a buried structure, which protects the electrical components from contact with harsh environments and reduces the resonant-frequency drift of the sensor in high-temperature environments. The pressure-sensitive membrane of the sensor is optimized according to small-deflection-plate theory, which allows the sensor to operate in high-pressure environments. The sensor is fabricated using low-temperature co-fired ceramic (LTCC) technology, and a fugitive film is used to create a completed sealed embedded cavity without an evacuation channel. The experimental results show that the frequency drift of the sensor *versus* the temperature is approximately 0.75 kHz/°C, and the responsivity of the sensor can be up to 31 kHz/bar within the pressure range from atmospheric pressure to 60 bar.

## 1. Introduction

The measurement of pressure parameters in harsh environments such as those experienced at high temperatures has become increasingly critical in automotive, aerospace, and industrial applications [1–3]. Despite the successful development of many pressure sensors relying on piezoresistance for high-temperature pressure monitoring, these sensors are based on silicon or silicon-on-insulator (SOI), which cannot operate in higher-temperature environments because the leakage current across the junctions changes drastically, and the sensor readings become invalid [4–7]. Other potential high-temperature materials such as silicon carbide have been reported, but their manufacturing processes are not as well developed as those for silicon [8]. To date, some pressure sensors based on low-temperature co-fired ceramic (LTCC) materials have been developed, but their performance is poor. For example, a high-temperature pressure sensor based on LTCC technology was designed and fabricated by the Georgia Institute of Technology in 2002, but the sensor has a large temperature drift in high-temperature environments, which will affect the accuracy for pressure testing in such environments [9,10]. In 2013, Xiong *et al.*, designed an improved LTCC-based capacitance pressure sensor, and the performance of the sensor is better than that of the aforementioned LTCC pressure sensor. However, the sensor can only measure pressures between 0 bar and 3.6 bar, and the temperature drift of the sensor is up to 10.5 kHz/°C [11].

In order to overcome the small pressure measurement range and large temperature drift, a high-performance embedded pressure sensor fabricated using LTCC technology by model design optimization is presented in this paper. The fabricated pressure sensor can be applied in extreme harsh environments, where an LC resonance circuit was embedded in a ceramic substrate. The sensitive membrane of the sensor is optimized so that the sensor can operate in high-pressure environments. A sealed cavity, inductance coil, and parallel capacitor plates are embedded into the ceramic substrate, which is fabricated using LTCC technology. Finally, the appropriate experimental test platforms are designed and set up to verify the performance of the fabricated sensor in high-temperature and high-pressure environments.

## 2. Measurement Principle and Optimal Design of the Sensor

**Figure 1.**
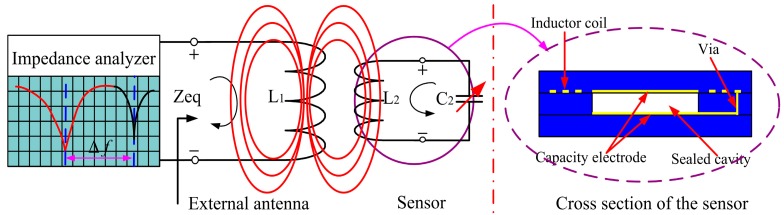
**(Left)** Conceptual schematic of noncontact wireless passive testing; **(Right)** Structural design principle of the sensor.

A diagram of the principle of the proposed pressure sensor is shown in Figure 1. A fixed inductance coil and variable capacitance are embedded into the ceramic substrate to form a series resonance circuit, and the deformable diaphragm can gauge pressure. When outside air pressure is applied to the sensitive membrane, the resonant frequency of the sensor will shift. The equivalent impedance *Z*_eq_ viewed from the testing antenna is expressed as follows, and the resonant frequency of the sensor can be derived [12,13]:

(1) $$Z_{eq}=j2\pi fL_{1}\left(1+\frac{k^{2}{\left(\frac{f}{f_{0}}\right)}^{2}}{1+\frac{j}{Q_{2}}\left(\frac{f}{f_{0}}\right)-{\left(\frac{f}{f_{0}}\right)}^{2}}\right)=F(f)$$

where *k* is the coupling coefficient, and *R*_1_, *f*, and *f*_0_ are the resistance, excitation frequency, and self-resonance frequency of the sensor, respectively. In addition, *Q*_2_ = (*L*_2_/*C*_2_)^1/2^/*R*_2_ is the LC resonance circuit quality factor of the sensor.

From Equation (1), we can conclude that the external reader antenna will build a stronger inductive coupling link with the sensor when *f* = *f*_0_. Moreover, the phase of the equivalent impedance from the testing antenna can be expressed as follows:

(2) ΔΦ=90−arctan(Im{Zeq}Re{Zeq})=tan−1(k2Q2)

From Equation (2), it could be concluded that the signal strength in the wireless sensing system can be increased by improving *k* and *Q*_2_, and the resonant frequency of the sensor will be detected clearly. For the inductance of the sensor, the electrical characteristics can be determined by using the established models, and the inductance of a planar square spiral coil is calculated as [14]:

(3) $$Ls=2.34\mu_{0}\frac{n^{2}(\frac{d_{\text{out}}+d_{in}}{2})}{1+2.75(\frac{d_{\text{out}}-d_{in}}{d_{\text{out}}+d_{in}})}$$

where *n* is the number of turns of the inductor coil, *μ*_0_ is the vacuum permeability, *d*_in_ is the inner diameter, and *d*_out_ is the outer diameter. The capacitance plates are designed for an embedded structure, and no ceramic dielectric exists between the two plates. Thus, the distance between capacitor plates is shortened, the capacitance of the sensor will increase, and the resonant frequency of the sensor will decrease, which is an advantage for signal collection in the low-frequency range. Further, the capacitance of the sensor under pressure can be described as:

(4) $$Cs=\frac{{ɛ}_{0}\pi a^{2}}{t_{g}}\cdot \frac{\tanh^{-1}(\sqrt{2d_{0}/t_{g}})}{\sqrt{2d_{0}/t_{g}}}$$

where *ε*_0_ is the permittivity of free space, *t*_g_ is the height of the embedded cavity, and *a* is the side length of the square electrode. When the edges of the membrane are fixed, the center deflection *d*_0_ of the membrane with applied pressure can be calculated from the following expression:

(5) $$d_{0}=\frac{3Pa^{4}(1-v^{2})}{16E{\left(t_{m}\right)}^{3}}\cdot \frac{1}{1+0.448{\left(\frac{d_{0}}{t_{m}}\right)}^{2}}$$

where *P* is the atmospheric pressure outside the sensor, and *E, t*_m_, and *v* are the Young modulus, the thickness of the membrane, and Poisson's ratio, respectively. When the deflection is small compared to the plate thickness (*d*_0_ ≪ *t*_m_), *d*_0_ can be expressed as:

(6) $$d_{0}≅\frac{3Pa^{4}(1-v^{2})}{16E{\left(t_{m}\right)}^{3}}$$

**Figure 2.**
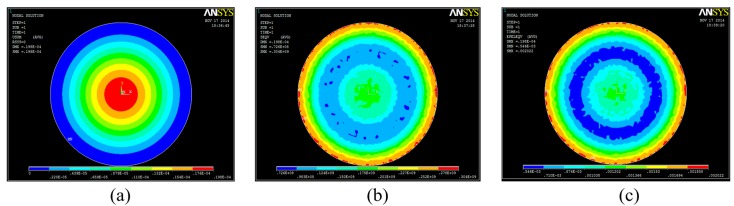
ANSYS simulation of the (**a**) deflection; (**b**) stress; and (**c**) strain of the sensitive membrane under a pressure of 60 bar.

From the aforementioned description, the quality factor of the sensor should be improved as much as possible in order to increase the signal strength between the sensor and the external antenna. The number of inductor coils is predetermined as 15, and the width of the inductance coil is increased as much as possible, which can increase the inductance, reduce the resistance, and improve the quality factor of the sensor. According to small-deflection-plate theory, an increase in the thickness of the sensitive membrane and a decrease in the sensitive membrane area would contribute to an improvement in the loaded pressure range. Therefore, the parameters of the sensor, including the capacitance-plate radius, the height of the embedded cavity, and height of the sensitive membrane were improved, and the specific parameters of the sensor are listed in Table 1. By simulation using ANSYS software, the deflection, stress, and strain of the sensitive membrane under 60 bar are shown in Figure 2.

## 3. Fabrication

**Figure 3.**
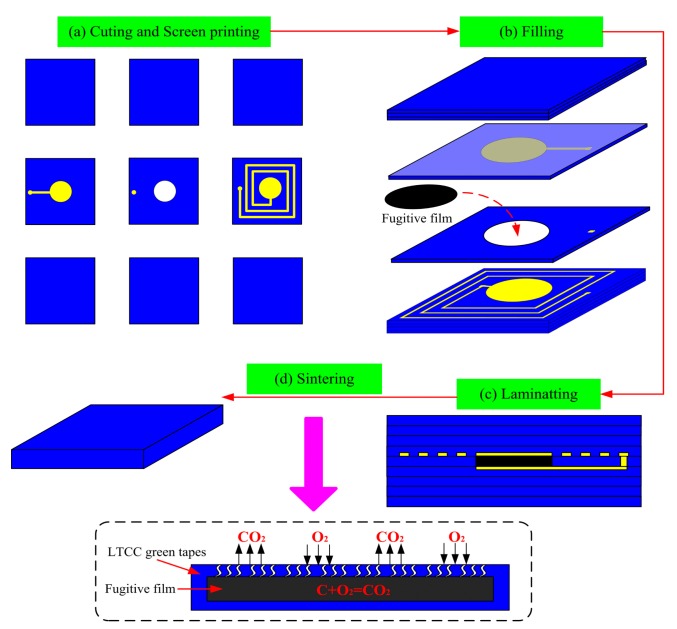
The sensor fabrication process.

The sensor is fabricated by LTCC technology, and the fabrication process is illustrated in Figure 3. Dupont 951 green tapes are cut into 8-inch square green tapes, and nine-layer green tapes are prepared for sensor fabrication.

### 3.1. Cutting and Printing

The fabrication process started with a pretreatment for greater than 30 min in an 80 °C drying oven. Then, the green tapes were cut to obtain accurate cavity, via, and alignment holes with the designed punch file using a punching machine. To achieve the embedded LC series resonance circuit on the substrate, the inductor and capacitor were screened-printed onto the green tapes using DuPont 6142 Ag conductor paste, and DuPont 6160 Ag conductor paste was used to fill the via hole to ensure that the circuit was connected, as shown in Figure 3a.

### 3.2. Filling

As shown in Figure 3b, the fugitive film was punched out at the same size as the diaphragm using the same punching file, and the punched cavity was filled with the fugitive film (the fugitive film will volatilize during co-firing, and the sealed cavity of the sensor will be formed).

### 3.3. Laminating

All layers were stacked together as the designed file to form a final stack. Then, the final stack was laminated in the laminating machine under a certain temperature and pressure to form a complete sensor sample, as shown in Figure 3c.

### 3.4. Sintering

**Figure 4.**
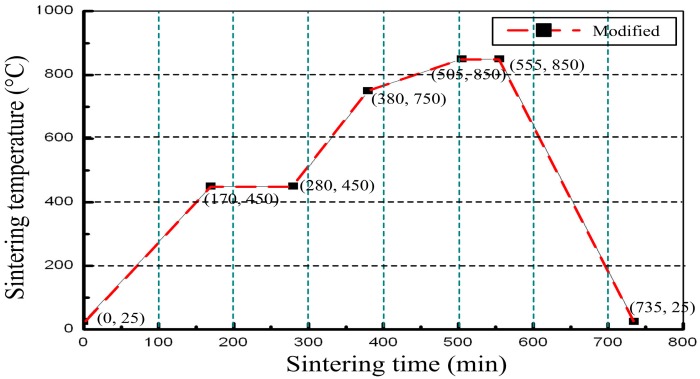
Temperature process control curve.

**Figure 5.**
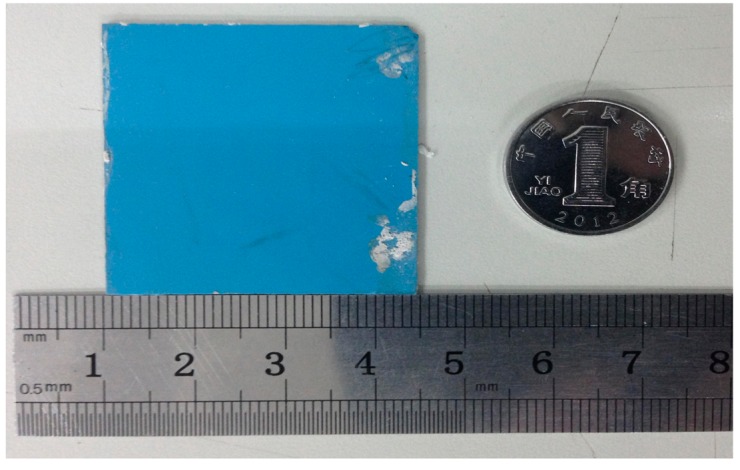
Fabricated embedded wireless passive pressure sensor.

The final stack was placed into a box furnace for sintering for approximately 110 min at 450 °C, which was beneficial to bake off the organics. The fugitive film is volatile around 800 °C, and at the same time, the ceramic is in the porous state. Therefore, gas components can be excluded during the reaction. The heating rate at 800 °C (approximately 0.8 °C/min ramp rate) was reduced in the sintering process, which can make the fugitive film fully burn with oxygen. The highest sintering temperature was 850 °C, and the dwell time of the highest temperature was approximately 30 min. Finally, the ceramic substrate completed the solid-phase reaction, and the fugitive film burned completely to form a sealed cavity, as shown in Figure 3d. In the sintering process, the furnace temperature was strictly controlled, and the specific sintering temperature curve is illustrated in Figure 4. An image of the fabricated embedded wireless passive sensor is shown in Figure 5.

## 4. Results and Discussion

The electrical parameters of the sensor were obtained by an E4991A impedance analyzer, and the experimental results are listed in Table 2. The values in Table 2 are approximately in good agreement with theoretical calculations.

**Figure 6.**
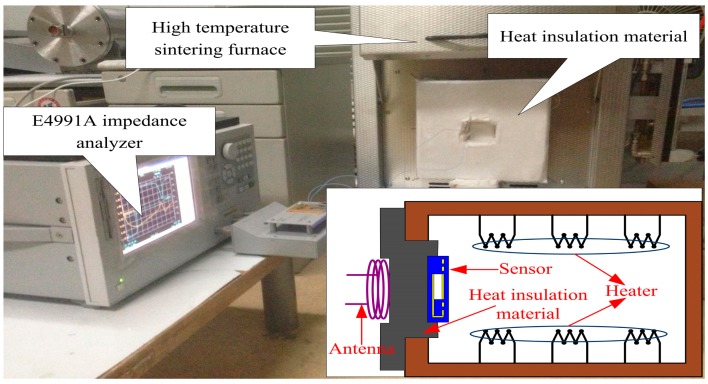
High-temperature measurement system.

High-temperature characterization of the sensor was conducted using a high-temperature sintering furnace to simulate a high-temperature environment. The temperature of the sintering furnace was controlled by a computer to ensure the heating rate and constant-temperature time. The sensor and antenna were separated by a distance through a heat insulation device, which is made of an inorganic nanometer refractory insulating material. Therefore, the sensor, which was placed in the furnace interior, can operate in high-temperature environments for signal collection, and the reader antenna, which was placed outside the furnace and connected to the E4991A Agilent impedance analyzer, can operate at room temperature for signal readout. The high-temperature measurement system is shown in Figure 6.

**Figure 7.**
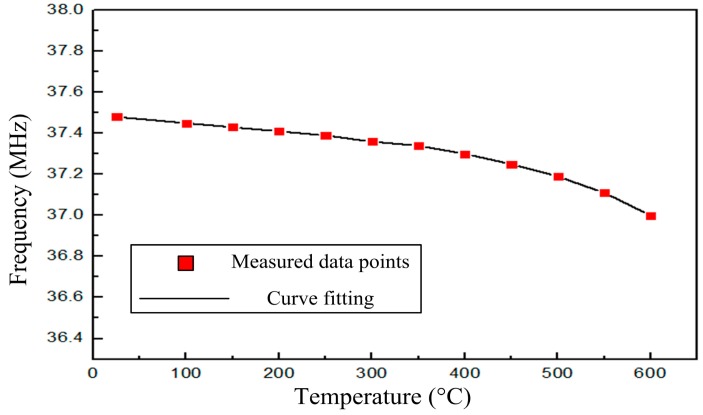
Measured resonant frequency of the sensor *versus* temperature.

Figure 7 shows the resonance frequencies of the sensor measured at atmospheric pressure from room temperature to 600 °C, and the resonance frequency of the sensor exhibits a slight change as the temperature increases. From the high-temperature performance test, we can conclude that the sensor can obtain a frequency signal in a high-temperature environment, which verifies the stability and availability of the sensor in high-temperature environments. Further, the average slope of the sensor is approximately −0.75 kHz/°C from room temperature to 600 °C, which is smaller than the previous LTCC pressure sensor studied by Xiong in 2013 [10]. In addition, the smaller frequency drift in a high-temperature environment will be more conducive to reducing errors caused at high temperature and obtaining a real pressure signal in high-temperature pressure environments.

**Figure 8.**
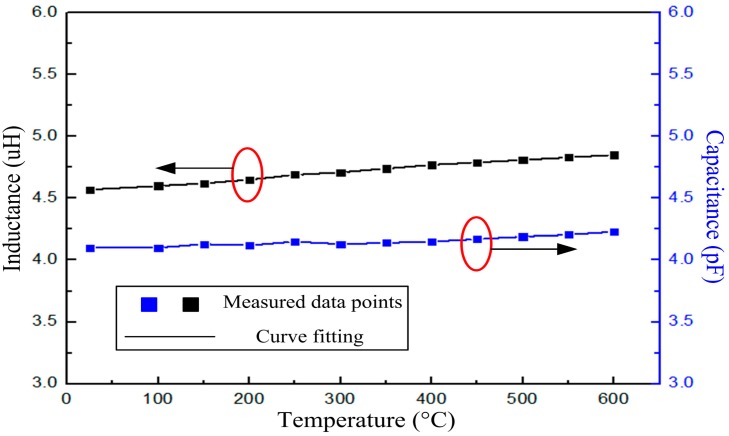
Measured inductance and capacitance *versus* temperature.

The inductor fabricated with the same structural parameters as the sensor was tested individually, and the inductance *versus* temperature is shown in Figure 8. According to the formula *f* = 1/(2π(*LC*)^1/2^), the capacitance of the sensor can be calculated from the measured resonant frequency and inductance, which is shown in Figure 8, from where we can conclude that the capacitance has little effect on the resonant frequency of the sensor. The reason is that the capacitance is designed to be embedded, and there is no ceramic dielectric between the capacitance plates. As the temperature increases, the relative permittivity has little effect on the capacitance in elevated temperature environments.

**Figure 9.**
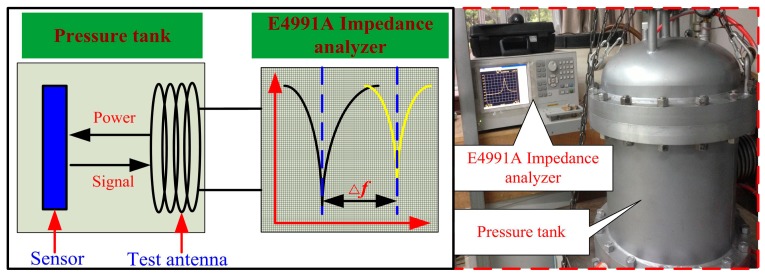
High-pressure measurement system.

**Figure 10.**
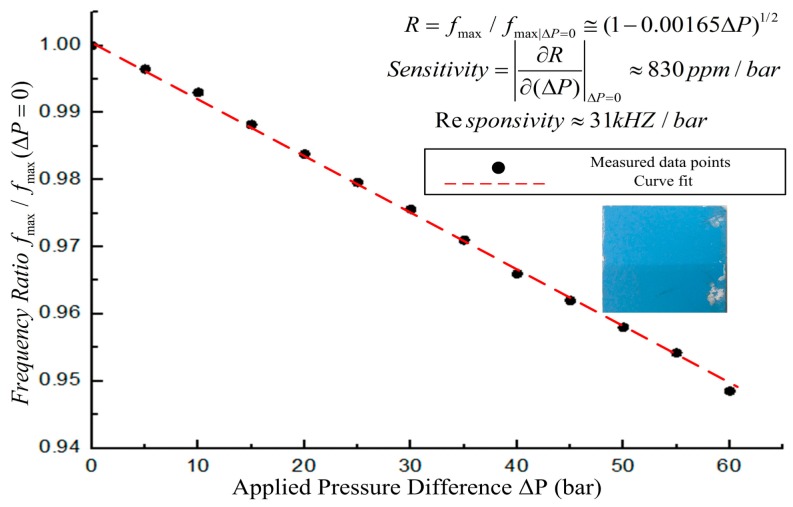
Pressure testing results of the sensor.

After the high-temperature characterization test, the sensor was tested to measure its pressure response using a high-pressure testing platform, which consists of an E4991A impedance analyzer and a pressure tank, as shown in Figure 9. In addition, the pressure can be controlled from atmospheric pressure up to 100 bar. The fabricated sensor was placed inside the testing system platform, and the antenna was connected to the E4991A impedance analyzer to complete the high-pressure testing. The normalized shifted resonant frequency of the sensor can be calculated from the following expression:

(7) $$\frac{f_{\max}(\Delta P)}{f_{\max}(\Delta P=0)}=\frac{\frac{1}{2\pi \sqrt{Ls(Cs+\Delta Cs)}}}{\frac{1}{2\pi \sqrt{LsCs}}}={\left(1-\alpha \Delta P\right)}^{1/2}=1-\frac{1}{2}\alpha \Delta P+\frac{1}{8}\alpha^{2}\Delta P^{2}-\cdot \cdot \cdot \cdot \cdot \cdot ≅1-\frac{1}{2}\alpha \Delta P$$

where Δ*C*_s_ is the change in capacitance due to diaphragm deflection, and *α* is a fitting parameter incorporating the mechanical behavior of the diaphragm. Thus, accurate environmental pressure variations could be created for the sensor (Δ*P* = *P*_outside sensor_ − *P*_inside sensor_) with this pressure control setup. Figure 10 shows the testing results of the sensor when the applied pressure is increased. From Figure 10, we can see that the sensitivity of the sensor is approximately 830 ppm/bar, and the responsivity of the sensor is approximately 31 kHz/bar within the pressure range from atmospheric pressure to 60 bar.

In the actual application environment, the application space of the sensor can be very small. To further reduce the size of the sensor, an embedded multi-layer-inductor sensor can be envisioned, where the inductance coil can be arranged in a three-dimensional coil to form a solenoid shape. Additionally, the high temperature ceramic can be used for the fabrication of the sensor, which can increase the working temperature of the sensor.

## 5. Conclusions

A high-performance noncontact wireless passive high-temperature pressure sensor has been successfully demonstrated in this paper. The structure of the sensor was optimized, which allows the sensor to operate in high-pressure environments and with a small temperature drift in high-temperature environments. A sealed cavity, inductance coil, and parallel capacitor plates were embedded into the ceramic substrate, which was fabricated using LTCC technology. The fabricated sensor was tested using high-temperature and high pressure system platforms, and the experimental results showed that the frequency drift of the sensor *versus* temperature is approximately 0.75 kHz/°C, which is far lower than previous LTCC pressure sensors. In addition, the responsivity of the sensor can be up to 31 kHz/bar within the pressure range from atmospheric pressure to 60 bar. In the future, the sensor will ultimately be tested in high-temperature pressure environments to faithfully register pressure variations in the automotive, aerospace, and aeronautics fields.

## Figures and Tables

**Table 1.** Parameters of the designed sensor.

**Symbol**	**Parameter**	**Value**
*a*	Radius of the capacitance plate metal	4 mm
*t*_g_	Height of the embedded cavity	110 μm
*t*_m_	Thickness of the sensitive membrane	440 μm
*d*_in_	Diameter of the inner inductor	9.8 mm
*d*_out_	Diameter of the outer inductor	22.5 mm
*n*	Number of coil turns	15
*l*_s_	Width of the coil turns	0.4 mm

**Table 2.** Electrical parameters of the sensor.

**Symbol**	**Parameters**	**Value**
*L*	Inductance	∼4.5 μH
*R*	Resistance	∼6.1 Ω
*C*	Capacitance	∼4.1 pF
*f*	Resonant frequency of the sensor	∼37.5 MHz
